# Enhancing Balance and Walking Endurance in Older Adults: The Potential of Transcranial Direct Current Stimulation as an Adjunct to Balance Training, a Randomized, Sham-Controlled, Clinical Trial

**DOI:** 10.3390/healthcare13111263

**Published:** 2025-05-27

**Authors:** Orathai Tunkamnerdthai, Panita Thamnithis, Chalermkiat Sawasdee, Keattichai Keeratitanont, Vichaya Auvichayapat, Wiyada Punjaruk, Somsak Tiamkao, Paradee Auvichayapat

**Affiliations:** 1 Department of Physiology, Faculty of Medicine, Khon Kaen University, Khon Kaen 40002, Thailand; 2 Non-Invasive Brain Stimulation Research Group of Thailand, Khon Kaen 40002, Thailand; 3 Department of Radiology and Nuclear Medicine, Faculty of Medicine, Burapha University, Chonburi 20131, Thailand; 4 Department of Physiology, Faculty of Medicine, Chulalongkorn University, Bangkok 10330, Thailand; 5 Department of Medicine, Faculty of Medicine, Khon Kaen University, Khon Kaen 40002, Thailand

**Keywords:** transcranial direct current stimulation, balance training exercise, older adults, walking endurance

## Abstract

**Background**: Falls among the elderly present significant physical, psychological, and economic challenges. Fall prevention strategies, such as balance and muscle strengthening exercises, are essential but often require long-term commitment. This study explores the potential of transcranial direct current stimulation (tDCS) as an adjunct to balance training to enhance physical performance in the elderly. **Method**: A randomized, double-blind, sham-controlled design was employed to compare balance training with active or sham tDCS. Participants underwent baseline assessments, followed by a six-week intervention period. The intervention protocol consisted of 2 mA, 20 min of anodal tDCS over the left primary motor cortex, three times weekly. Post-intervention assessments were conducted a few days after the intervention and follow-up at 4 weeks. **Results**: Following 18 sessions of anodal tDCS combined with balance exercise training, no significant group differences were observed for the Time Up and Go, One-Leg Standing, lower-limb strength, or the 6 min walk test (6MWT), although both the intervention and control groups demonstrated significant improvements over time. A significant group × time interaction was found only for the 6MWT, with participants in the intervention group exhibiting greater improvements in the 6MWT compared to controls. **Conclusions**: Anodal tDCS combined with balance exercise training selectively enhanced physical endurance but did not confer additional benefits for balance, gait, or leg strength in healthy older adults. These findings suggest that tDCS may serve as a promising adjunct to exercise for improving endurance-related outcomes in aging populations. Control of various variables for tDCS and exercise is necessary.

## 1. Introduction

Falls among older adults pose significant and multifaceted consequences. Physically, falls can lead to injuries ranging from minor lacerations to severe fractures [1]. Psychologically, the experience of falling often induces fear and anxiety, resulting in diminished confidence in mobility [2]. Economically, fall-related injuries frequently require medical intervention and extended hospitalizations, contributing substantially to healthcare expenditures [3].

Exercise training remains a cornerstone of fall prevention strategies. Effective programs typically incorporate balance training activities, such as single-leg stands, tandem walking, and figure-eight walking, to enhance stability [4].

Muscle strengthening exercises, including squats, toe stands, and heel stands, further improve lower-limb strength [5], while flexibility exercises targeting the ankles and trunk augment overall mobility [6]. Evidence suggests that engaging in balanced exercises at least two times per week over a minimum of three months significantly reduces fall risk [7]. However, such prolonged training periods can be challenging for older adults to sustain, highlighting the need for adjunctive strategies to enhance training efficiency and outcomes.

Transcranial direct current stimulation (tDCS) is a non-invasive neuromodulation technique characterized by its ease of use and favorable safety profile. Scientific evidence indicates that tDCS enhances motor training through three principal mechanisms: (1) modulation of cortical excitability to promote neuroplasticity and consolidate motor learning [8]; (2) augmentation of synaptic plasticity to improve retention of motor skills [9]; and (3) increased neural network efficiency to facilitate the integration of new motor patterns [10]. Priming the motor cortex with anodal tDCS before exercise may, therefore, create an optimal neural state, promoting more effective motor learning and performance gains.

A meta-analysis of 10 randomized controlled trials (RCTs) reported that only 2 studies combined tDCS with physical training [11,12], while the remaining 8 administered tDCS alone [13]. Most trials involved a single tDCS session administered to healthy older adults [14,15,16,17,18,19]. Only one study in healthy older adults delivered multiple sessions (specifically, 6 sessions of tDCS [12]), whereas studies in clinical populations reported longer interventions (e.g., 16 sessions in stroke patients [20] and 10 sessions in individuals with slow gait and cognitive impairments [21]).

The meta-analysis, which included 280 participants, revealed that tDCS significantly improved balance control with low heterogeneity. Substantial effects were observed in outcomes, such as the Timed Up and Go (TUG) test, the Berg Balance Scale, postural sway measures, and walking speed under dual-task conditions (standardized mean differences [SMDs]: −0.99 to 3.41; 95% confidence limits [CL]: −1.52 to 4.50; *p* < 0.006; I^2^ < 52%). Moderate to large effects were also noted in postural stability assessments on static and movable platforms (SMDs: 0.37 to 1.12; 95% CL: −0.09 to 1.62; *p* < 0.03; I^2^ < 62%) [13].

Previous RCTs have demonstrated that five sessions of anodal tDCS, delivered at 1 mA for 20 min over the primary motor cortex (M1) without concurrent exercise, significantly improved TUG performance in healthy older adults [22]. Furthermore, improvements in maximum force exertion during walking tests were evident one week after the final session.

Despite these promising findings, studies in healthy older adults have typically employed limited numbers of tDCS sessions and measured outcomes within short follow-up periods. Recent work by Guo et al. (2024) demonstrated that multisession tDCS could induce lasting motor improvements [23]. Given that physical exercise alone enhances neurotransmitter and neurotrophic factor synthesis, promotes neurogenesis, and facilitates neuroplasticity [24,25], the combination of exercise and tDCS may synergistically amplify cortical excitability and motor learning.

Building upon this body of evidence, we hypothesized that increasing the number of tDCS sessions to 18, combined with balance exercise training, would produce durable improvements in physical performance among healthy older adults. Therefore, the aim of the present study was to investigate the effects of six weeks of balance exercise training combined with anodal tDCS (18 sessions in total) on static balance, dynamic balance, lower-limb muscle strength, and physical endurance, with follow-up assessments conducted 4 weeks post-intervention.

## 2. Materials and Methods

### 2.1. Study Design

**Figure 1:**
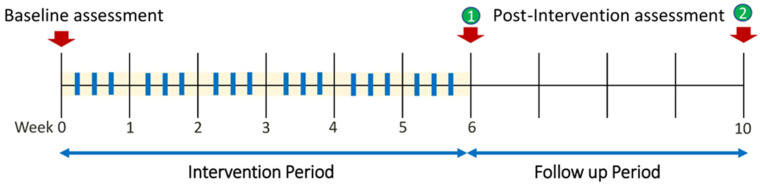
Timeline of the study.

This study employed a randomized, double-blind, sham-controlled design to compare two intervention conditions: balance training combined with sham transcranial direct current stimulation (tDCS) and balance training combined with active tDCS. The study protocol was divided into three distinct phases: Baseline Assessment: Participants underwent initial evaluations, including the TUG test, One-Leg Standing Test (OLST), lower-limb strength assessment, and the Six-Minute Walk Test (6MWT). Intervention Period: A few days after the baseline assessments, participants commenced their assigned treatment to support physical recovery. Depending on randomization, they received either sham or active tDCS combined with supervised exercise training three times per week for six consecutive weeks. The six-week duration was selected based on prior research, demonstrating that this timeframe is sufficient to induce meaningful neuroplastic adaptations and improvements in motor function [26,27]. Follow-up Period: Participants were re-evaluated using the same outcome measures at two post-intervention time points, first, 2–3 days after completing the six-week training program, and second, four weeks after the intervention period (Figure 1).

### 2.2. Participant Recruitment and Informed Consent

Sample size calculation was performed using G*Power 3.1.9.7 software based on a randomized controlled trial design. The required sample size was determined using the Modified Figure of 8 Walk Test Time variable, as reported by Rostami et al. (2020) [22], to achieve a statistical power of 80% and a two-sided significance level of 0.05. Based on these parameters, the estimated sample size was 30 participants. To account for potential attrition due to the extended follow-up period, an additional 20% was added, resulting in a final target of 36 participants (18 per group).

**Figure 2:**
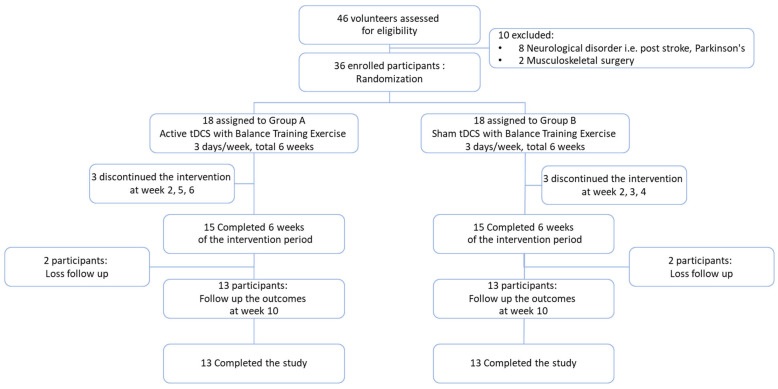
Flow of participants.

Participants were recruited via community announcements in Khon Kaen, Thailand, between 20 June 2023 and 15 November 2023. A total of 46 volunteers were screened for eligibility; 10 were excluded due to neurological disorders or previous musculoskeletal surgery, resulting in 36 participants being enrolled. Participants were randomized into two groups: Group A: Active transcranial direct current stimulation (tDCS) combined with balance training exercises (n = 18). Group B: Sham tDCS combined with balance training exercises (n = 18). Both interventions were administered three times per week over a six-week period. In Group A, 3 participants discontinued at weeks 2, 5, and 6, resulting in 15 participants completing the six-week intervention. Two additional participants were lost to follow-up at week ten, leaving thirteen participants who completed this study. Similarly, in Group B, three participants discontinued at weeks 2 and 4, and two participants were lost at follow-up, also resulting in thirteen participants completing this study. The participant flow is detailed in Figure 2.

#### Eligibility Criteria

Healthy elderly participants were defined according to the World Health Organization’s 2020 criteria for healthy aging and functional ability, which includes being free from major chronic diseases or conditions that significantly impair daily functioning, being able to independently perform activities of daily living, and maintaining a good level of cognitive function [28]. Screening was conducted by a physician (PA). Inclusion criteria included the following: (a) age 65 years or older; (b) ability to walk independently for at least 10 m without the use of a walking aid; (c) ability to rise from a chair with or without arm support; (d) ability to understand and follow instructions for the study tasks. Exclusion criteria included the following: (a) severe medical conditions such as advanced cardiovascular, pulmonary, or neurological disorders that may affect balance; (b) use of medications known to impact balance (e.g., causing dizziness or weakness); (c) musculoskeletal diseases, injuries, or surgeries within the past six months; (d) severe psychiatric conditions that could interfere with participation; (e) participation in concurrent research studies involving balance training or similar exercise regimens; (f) presence of metallic implants near the stimulation site; (g) use of a pacemaker; (h) presence of cranial bone defects.

**Ethics Approval:** This study was conducted in accordance with the principles outlined in the Declaration of Helsinki. Ethical approval was obtained from the Ethics Committee of Khon Kaen University (approval number HE651097). The trial was registered with the Thai Clinical Trials Registry (TCTR20230614002) on 14 June 2023 prior to the enrollment of the first participant (https://www.thaiclinicaltrials.org/show/TCTR20230614002 (accessed on 14 June 2023)).

**Informed Consent:** Informed consent was obtained from all participants prior to their inclusion in this study. Participants received detailed information regarding the study’s objectives, procedures, potential risks, and benefits. They were explicitly informed of their right to withdraw from the study at any time without facing any consequences. Additionally, participants were assured that their data would remain confidential and would be used exclusively for research purposes.

**Confidentiality of Participant Data**

All participant data were managed with the utmost confidentiality. Data were securely stored, with access strictly limited to authorized personnel. Personal identifiers were removed to ensure participant privacy, in compliance with the confidentiality protocols established by the Ethics Committee of Khon Kaen University.

### 2.3. Randomization and Blinding

Following informed consent and completion of baseline assessments, each participant was assigned a sequential identification number by the study manager. The study manager provided the statistician with participants’ results from the TUG test, OLST, lower-limb strength test, 6MWT, and age. A computerized randomization schedule was generated by the statistician to balance the TUG results across treatment conditions. Participants were randomly assigned to either Group A or Group B in a 1:1 ratio. All outcome assessors were blinded to the clinical context of each participant and to their assigned treatment group. Participants were instructed to continue their routine medication regimen throughout this study. The personnel responsible for generating the random allocation sequence, enrolling participants, and assigning interventions had no involvement in assessments, and all assessors were blinded to the treatment allocation.

### 2.4. Transcranial Direct Current Stimulation

Transcranial direct current stimulation (tDCS) was delivered using wet electrode pads (5 × 7 cm) and a battery-powered device (NeuroKK™, Khon Kaen, Thailand), with a maximum output of 2 mA. Anodal stimulation was applied to the left primary motor cortex (M1), which is implicated in motor function-related neuronal plasticity [13], according to the 10–20 system of electrode placement. The cathode was positioned on the right shoulder to minimize interference from the reference electrode and enhance the focality of cortical modulation [29]. The stimulation protocol consisted of three sessions per week for six weeks. After a 30 s ramp-up phase, the current was maintained at 2 mA for 20 min, followed by a ramp-down phase. The device was capable of delivering both active and sham stimulation. In the sham condition, the current was discontinued after 30 s, while the power indicator remained illuminated to ensure blinding, as the indicator was visible to participants during both active and sham conditions. tDCS was administered prior to balance exercises in each session.

### 2.5. Balance Exercise Training

After completing the tDCS session, participants engaged in balance exercise training for 40 min per session, three sessions per week, over a total of six weeks. Each balance exercise training session consisted of three components: (1) Warm-up (5 min): Participants performed marching in place, head movements, neck movements, back extensions, trunk movements, and ankle movements. (2) Balance exercise session (30 min): Participants completed a series of balance exercises, including knee bends, toe walking, heel–toe standing, heel–toe walking, one-leg stands, sideways walking, heel walking, sit-to-stand transitions, backward walking, heel–toe walking backward, and walk-and-turn tasks. They were allowed to perform the exercises with or without external support, depending on individual needs. (3) Cool-down session (5 min): Participants concluded with calf stretching and posterior thigh (hamstring) stretching.

## 3. Measures

### 3.1. Assessment of the Timed up and Go Test

The Timed Up and Go (TUG) test was utilized as the primary outcome measure to assess mobility, balance, gait ability, and fall risk in older adults. The TUG is a reliable, simple, and cost-effective method for evaluating activities of daily living (ADL) [30,31]. It demonstrates excellent reliability, with an intraclass correlation coefficient (ICC) of 0.99. To perform the TUG, participants begin seated in a chair (46 cm in height) with their back against the chair. Upon the command “go”, they stand up, walk forward 3 m at a comfortable pace, turn around, walk back to the chair, and sit down. The timing starts at the “go” command and ends when the participant is seated again. Participants were allowed one practice trial, which was excluded from the final assessment. Following a 15 min rest, one formal trial was conducted [32]. A shorter completion time indicates better mobility, balance, and gait function [33]. Clinically, participants completing the test in 10–19 s are typically independent, with adequate balance and walking speed, while those requiring 30 s or more may experience significant dependence in daily activities [34]. The assessment was conducted following the method outlined by Podsiadlo and Richardson (1991) [30].

### 3.2. Assessment of the One-Leg Standing Test

The One-Leg Standing Test (OLST) is an established tool for assessing static balance and fall risk, with excellent reliability (ICC > 0.9) [35,36]. In our study, OLST was used as a secondary outcome measure. Participants performed the test alternately on each leg, standing unshod. They were instructed to raise one leg while flexing the contralateral knee at a 90° angle, keeping the thigh parallel to the standing leg. The duration of the one-leg stance was measured using a stopwatch, with a 10 s rest between trials. If participants were unable to hold the position for 30 s, up to three attempts were allowed, and the longest duration was recorded. The total OLST time was the sum of both legs’ performance [36]. Longer durations indicate better balance and lower fall risk, while failing to maintain the position for at least 5 s is a predictor of future falls. Holding the stance for at least 10 s is associated with reduced all-cause mortality [37]. The assessment adhered to the procedure outlined by Khanal et al. (2021) [36].

### 3.3. Assessment of Lower-Limb Strength

Lower-limb strength was assessed using a back and leg dynamometer (TKK 5002; Takei Scientific Instruments Co., Niigata, Japan) to measure isometric force, demonstrating an ICC > 0.91 [38]. Participants stood on the dynamometer base with slight knee and hip flexions while maintaining a lordotic curve in the lower back. They performed vertical lifts through continuous isometric contractions of the knee and hip extensors. Three peak force measurements were taken, with each contraction lasting 5 s and a 60 s rest interval between measurements. Verbal encouragement and stabilization were provided to ensure accurate results [39]. Higher peak force values indicate greater leg strength, which is clinically linked to better functional performance and a reduced risk of mobility-related issues. The assessment followed the methodology described by Coldwells et al. (1994) [39].

### 3.4. Assessment of the Six-Minute Walk Test

The Six-Minute Walk Test (6MWT) was used to assess physical endurance and functional capacity, following the American Thoracic Society guidelines. It demonstrates strong reliability, with an ICC ranging from 0.86 to 0.96 [40,41]. Participants were instructed to walk as far as possible within 6 min, traversing back and forth around a cone placed 10 m apart. The assessor provided safety supervision and time updates every minute. Participants were allowed to rest, if necessary, but the timing continued uninterrupted. Longer distances walked suggest better physical endurance and cardiovascular fitness. A 30 m or 7–9% increase in the 6MWT distance is considered clinically significant in patients with chronic conditions such as heart failure or pulmonary disease [42]. The 6MWT was conducted in accordance with Cazzoletti et al. (2022) [43].

### 3.5. Statistical Analysis

Statistical analyses adhered to the intention-to-treat principle. Descriptive statistics, including means and standard deviations, were calculated for demographic and outcome variables. The normality of the data was assessed using the Shapiro–Wilk test. The primary analysis focused on comparing static balance ability (TUG) and dynamic balance ability (OLST). Secondary outcomes included lower-limb muscle strength, and tertiary outcomes involved physical endurance (6MWT). Repeated measures analysis of variance (ANOVA) was employed to evaluate differences over time, between groups, and the interaction between group and time points. Within-group differences across each time point were also assessed using repeated measures ANOVA. When significant effects were detected, post hoc comparisons were conducted using Tukey’s Honest Significant Difference (HSD) test. The post hoc analysis of within-group differences focused solely on ‘Baseline-Post Intervention’ and ‘Baseline-Follow-up’. Effect sizes were calculated using Cohen’s d, with 0.2, 0.5, and 0.8 indicating small, medium, and large effect sizes, respectively. Statistical analyses were performed using SPSS Statistics version 21, with a significance threshold set at *p* < 0.05. KK and VA supervised data quality and accuracy throughout the analysis.

### 3.6. Adverse Events

Although tDCS involves the application of modest direct current, participants were asked to report any adverse events or symptoms immediately following each stimulation session to ensure safety. Physicians closely monitored participants during the trial and checked for negative side effects seven days post-procedure.

## 4. Results

### 4.1. Demographic and Baseline Characteristics

Participants in both groups had similar demographic profiles. The mean age was 71.73 years in Group A and 74.87 years in Group B. The mean BMI was 23.84 kg/m^2^ for Group A and 23.03 kg/m^2^ for Group B. Group A consisted of 1 male and 14 females, while Group B had 5 males and 10 females. The mean waist and hip ratio was 0.90 in Group A and 0.91 in Group B. Regarding medication use, 46.67% of Group A and 33.33% of Group B participants took no medications. The distribution of participants taking 1, 2, and ≥3 medications was similar across both groups. Underlying diseases included diabetes mellitus, hypertension, hyperlipidemia, and other conditions, with comparable prevalence between the groups (Table 1).

### 4.2. Timed up and Go Test

**Figure 3:**
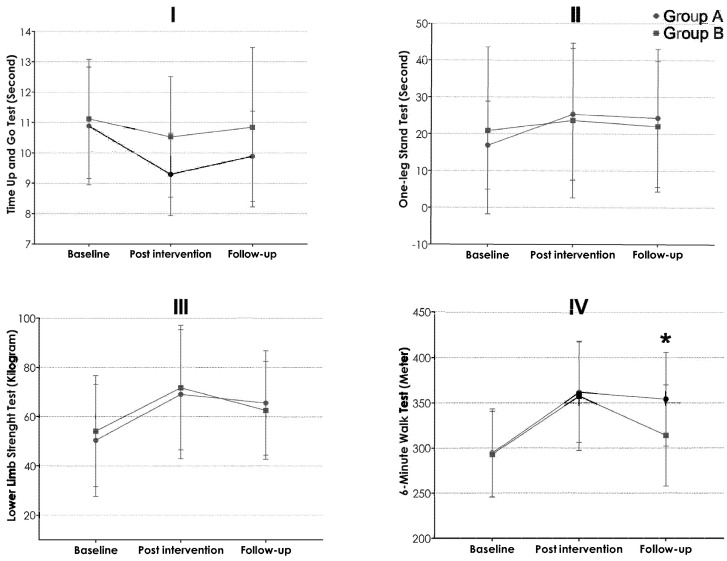
The mean outcomes of the Timed Up and Go test (**I**), One-Leg Standing Test (**II**), lower-limb strength test (**III**), and Six-Minute Walk Test (**IV**). The study cohorts comprised Group A, which underwent five sessions of active transcranial direct current stimulation (tDCS), and Group B, which received five sessions of sham tDCS thrice weekly, coupled with balance exercises over a six-week period. An asterisk (*) denotes *p* < 0.05, indicating statistical significance between the groups. The error bars represent standard deviation (SD) as determined by ANOVA.

A repeated-measures ANOVA, with group as a between-subjects factor and time as a within-subjects factor, revealed no significant main effect of group (F(1, 28) = 1.51, *p* = 0.230). However, there was a significant main effect of time (F(2, 56) = 11.91, *p* < 0.0001, η^2^ = 0.298), indicating that the TUG scores improved over time. The interaction between time and group was not statistically significant (F(2, 56) = 2.65, *p* = 0.079, η^2^ = 0.087) (Figure 3I). Post hoc analysis showed that both Group A and Group B exhibited significant improvements in their mean TUG scores. Group A demonstrated a statistically significant improvement (*p* = 0.001), with a very large effect size (Cohen’s d = 1.03). Group B also showed a statistically significant improvement, though with a medium effect size (Cohen’s d = 0.32).

In the long-term analysis, conducted four weeks post-intervention during the follow-up period, there was a trend towards continued improvements in Group A (*p* = 0.092). However, Group B did not exhibit statistically significant changes. In summary, while both groups demonstrated significant improvements in TUG scores, Group A displayed a larger effect size and a trend towards superior long-term outcomes compared to Group B.

### 4.3. One-Leg Standing Test

There was no significant main effect of group F(1, 28) = 0.000, *p* = 0.998). However, there was a significant main effect of time (F(2, 56) = 5.88, *p* = 0.005, η^2^ = 0.174). The interaction between time and group was not statistically significant F(2, 56) = 2.04, *p* = 0.139, η^2^ = 0.068). Post hoc analysis revealed that both Group A and Group B showed significant improvements in mean OLST scores. Group A demonstrated statistically significant improvements (*p* = 0.009), with a medium effect size (0.60). Group B also showed statistically significant improvements (*p* = 0.041), with a small effect size (0.14). In the long-term analysis, conducted four weeks post-intervention, or the follow-up period, Group A showed a trend towards significant differences (*p* = 0.061), whereas Group B did not exhibit statistically significant differences. In the overall OLST outcomes, both Group A and Group B showed improvements in OLST scores, with Group A demonstrating a larger effect size and a trend towards better long-term outcomes compared to Group B.

### 4.4. The Lower-Limb Strength Test

A repeated-measures ANOVA with group as a between-subjects factor and time as a within-subjects factor revealed no significant main effect of group (F(1, 28) = 0.000, *p* = 0.998). However, there was a significant main effect of time (F(2, 56) = 5.88, *p* = 0.005, η^2^ = 0.174), indicating significant changes in OLST scores over time. The interaction between time and group was not statistically significant (F(2, 56) = 2.04, *p* = 0.139, η^2^ = 0.068).

Post hoc analysis revealed that both Group A and Group B showed significant improvements in mean OLST scores. Group A demonstrated a statistically significant improvement (*p* = 0.009), with a medium effect size (Cohen’s d = 0.60). Group B also showed statistically significant improvements (*p* = 0.041), albeit with a small effect size (Cohen’s d = 0.14).

In the long-term analysis, conducted at the four-week post-intervention follow-up, Group A showed a trend towards significant differences (*p* = 0.061), whereas Group B did not exhibit statistically significant changes. Overall, both groups demonstrated improvements in OLST scores, with Group A showing a larger effect size and a trend towards superior long-term outcomes compared to Group B.

### 4.5. The Six-Minute Walk Test

A repeated-measures ANOVA with group as a between-subjects factor and time as a within-subjects factor revealed no significant main effect of group (F(1, 28) = 0.77, *p* = 0.389). However, a significant main effect of time was observed (F(2, 56) = 36.31, *p* < 0.001, η^2^ = 0.565), indicating that both groups exhibited substantial improvements over time. Importantly, the interaction between time and group was statistically significant (F(2, 56) = 3.88, *p* = 0.026, η^2^ = 0.122), highlighting differential changes in 6MWT scores between the two groups. Post hoc analysis showed that both Group A and Group B exhibited significant improvements in mean 6MWT scores. Group A demonstrated a statistically significant improvement (*p* < 0.001), with a very large effect size (Cohen’s d = 1.37). Group B also showed a statistically significant improvement (*p* < 0.001), with a very large effect size (Cohen’s d = 1.27).

In the long-term analysis, conducted at the four-week post-intervention follow-up, Group A continued to show statistically significant improvements (*p* < 0.001), with a very large effect size (Cohen’s d = 1.26), whereas Group B did not exhibit statistically significant differences (*p* = 0.350). The post hoc analysis revealed a trend indicating a significant difference during the follow-up period in Group A. In addition, the mean 6-MWT distance in Group A was 40.2 m greater than in Group B. This difference is considered clinically significant, suggesting that Group A exhibited better physical endurance and cardiovascular fitness compared to Group B [42]. Comparative analysis of outcomes between Group A and Group B over the same timepoints are presented in Table 2. The differential outcomes within the group observed at baseline, post-intervention, and follow-up are presented in Table 3. 

In summary, both Group A and Group B showed significant improvements in 6MWT outcomes. However, Group A demonstrated a larger effect size and better long-term outcomes compared to Group B, suggesting that balance training combined with active tDCS has a more substantial long-term impact on physical endurance, gait ability, and fall risk reduction in healthy elderly individuals compared to balance training combined with sham tDCS.

### 4.6. Adverse Events

All participants completed the experiment with no severe adverse effects. Two participants in Group A reported transient tingling and five participants in Group A reported itching under the electrodes.

## 5. Discussion

The present study investigated the long-term effects of anodal tDCS combined with balance exercise training on balance, gait ability, leg muscle strength, and physical endurance in healthy older adults. Following 18 sessions of anodal tDCS over six weeks, participants were assessed using the TUG test, OLST, lower-limb strength tests, and the 6MWT. The findings revealed no significant main effect of group, indicating comparable overall outcomes between the intervention and control groups. However, a significant main effect of time was observed across all measures, suggesting that participants in both groups improved during the intervention period. Notably, no significant group × time interaction was found for the TUG, OLST, or lower-limb strength tests, whereas a significant interaction emerged for the 6MWT. Post hoc analyses demonstrated that participants in the intervention group exhibited significantly greater improvements in 6MWT performance compared to the control group. These results suggest that anodal tDCS, when combined with balance training, may selectively enhance long-term physical endurance and functional capacity beyond the effects of exercise alone.

The role of tDCS as an adjunct to physical training has garnered increasing attention due to its potential to enhance physical performance across diverse populations. Here, we contextualize our findings alongside previous studies investigating the combined effects of tDCS and exercise on balance, gait, leg strength, and endurance, particularly in healthy older adults and clinical populations.

### 5.1. Synergistic Effects of tDCS and Exercise on Balance and Lower-Limb Muscle Strength

With respect to balance, gait ability, and leg strength, our study demonstrated improvements in both the tDCS and sham groups, without additional benefit from tDCS. These findings align with those of Sánchez-Barbadora et al. (2025), who similarly reported no significant advantage of combining tDCS with balance training in young adults [44]. One plausible explanation for these results is the presence of a ceiling effect associated with intensive exercise training, as discussed by Vergallito et al. (2022) [45]. Exercise alone is well established to induce beneficial structural and functional brain changes, including enhanced neurogenesis, synaptogenesis, angiogenesis, and increased levels of neurotrophic factors such as brain-derived neurotrophic factor (BDNF) [46]. Importantly, sustained physical activity over several weeks is necessary for new neurons to mature and integrate functionally into existing networks [47]. At the molecular level, exercise promotes neuroplasticity through the upregulation of BDNF, insulin-like growth factor-1 (IGF-1), modulation of neurotransmitter systems, and epigenetic mechanisms [48]. Given these extensive neuroplastic adaptations induced by exercise alone, it is plausible that long-term potentiation (LTP)-like plasticity within the motor system had already reached near-saturation. As proposed by Stagg and Nitsche (2011), when endogenous neural plasticity is saturated—a phenomenon known as the ceiling effect—the capacity for further modulation through interventions such as anodal tDCS may be diminished or masked [10].

Beyond the ceiling effect, methodological and participant-related factors may have contributed to the lack of additional tDCS benefit observed. First, age-related reductions in synaptic plasticity and cortical excitability may limit responsiveness to neuromodulation in older adults [49,50]. Studies involving younger participants (e.g., Song and Yim, 2023) have reported more robust responses to tDCS [51], likely reflecting greater baseline neuroplastic potential. Second, the electrode montage used in our study—placing the cathode extracephalically over the right shoulder—differs from the more conventional supraorbital placement (e.g., Yosephi et al. 2018; Mehrdadian et al. 2023) [12,52]. As highlighted by Thair et al. (2017), electrode placement critically affects the direction and intensity of current flow in the brain; thus, our montage may have attenuated stimulation intensity over targeted cortical areas [29]. Third, the timing of stimulation may have influenced outcomes: we applied tDCS in an offline mode (prior to exercise), whereas several positive studies delivered stimulation concurrently during motor tasks (online mode) [12,51,52], potentially enhancing task-specific plasticity [10,45]. Finally, while our study involved a relatively large number of sessions [18], increasing the opportunity for long-term adaptation, it may also have introduced greater interindividual variability, particularly given that factors such as brain anatomy, motivation, and attention are known to modulate tDCS efficacy [45]. Together, these findings emphasize the need for individualized tDCS protocols, carefully optimized for age, electrode configuration, stimulation timing, and intervention duration.

### 5.2. Synergistic Effects of tDCS and Exercise on Endurance

In terms of endurance, our study demonstrated that anodal tDCS applied before balance training significantly enhanced endurance performance, as measured by the 6MWT, in healthy older adults compared to controls. To our knowledge, this is the first study to specifically investigate the effects of combined tDCS and exercise on endurance outcomes in this population. Although direct comparisons are limited, improvements in walking speed—a related component of endurance—have been reported by Massaferri et al. (2023) in chronic stroke patients following combined tDCS and multimodal physical training (MPT) [53]. For instance, a 12-week intervention involving 2 mA anodal tDCS during exercise sessions resulted in significant gains in both walking speed and cardiorespiratory performance [53]. These findings support the hypothesis that tDCS may potentiate exercise-induced adaptations in populations with compromised neuroplasticity. The mechanisms underlying these improvements likely involve enhanced cortical excitability, promoting greater neural adaptation to training stimuli. Anodal tDCS applied over the primary motor cortex (M1) has been shown to facilitate motor learning and strengthen motor pathways, effects that may be particularly beneficial in populations where plasticity is diminished, such as older adults and individuals with neurological impairments [54]. By contrast, studies in younger, healthier populations have reported less consistent effects. Jung et al. (2024) found that physical training alone, but not tDCS combined with training, significantly improved endurance scores in healthy young adults [55]. Similarly, Yang et al. (2024) observed no enhancement in aerobic endurance following tDCS prior to exercise in elite swimmers, although gains in strength and specific performance metrics were noted [56]. These findings suggest that in populations operating near physiological limits, the additive benefits of tDCS may be minimal, and the optimization of stimulation parameters may be necessary to observe measurable gains.

Taken together, these results indicate that tDCS can serve as a valuable adjunct to physical training, particularly in populations characterized by diminished motor function or neuroplasticity, such as older adults and clinical populations. However, the efficacy of tDCS appears to be highly population-specific, influenced by baseline plasticity levels, the type of exercise performed, and stimulation parameters. Future research should prioritize individualized approaches to tDCS application, with careful attention to subject characteristics, training regimen, electrode configuration, and stimulation timing, to optimize therapeutic outcomes.

## 6. Implications, Limitations, and Future Directions

The findings of the present study have important implications for the use of neuromodulatory interventions in older adults. Although the addition of anodal tDCS to balance exercise training did not further enhance balance, gait, or leg strength beyond the effects of exercise alone, the observed improvement in endurance performance suggests that tDCS may selectively augment certain domains of physical function. These results highlight the potential role of tDCS as an adjunctive strategy to amplify exercise-induced benefits, particularly in aspects related to aerobic capacity and functional endurance. Importantly, the outcomes underscore the complexity of neuroplastic responses in aging populations and the necessity of tailoring interventions to individual neurophysiological profiles. Clinicians and researchers should recognize that the efficacy of combined tDCS and exercise protocols may vary across different functional outcomes and that careful consideration of stimulation parameters and population characteristics is critical for optimizing therapeutic effects.

Future research should prioritize the development of personalized tDCS protocols, optimizing factors, such as electrode montage, stimulation timing (online vs. offline application), intensity, and session frequency, to maximize synergistic effects with exercise interventions. Studies involving real-time (online) stimulation during motor tasks may further elucidate whether task-specific plasticity can be more effectively enhanced in older adults. Additionally, investigations targeting clinical populations characterized by impaired motor function or reduced neuroplasticity, such as individuals with neurological disorders, may reveal broader applications of combined tDCS and exercise approaches. Longitudinal studies with extended follow-up periods are warranted to assess the durability of functional gains and to explore whether booster sessions of tDCS could sustain or further amplify training-induced improvements. Finally, incorporating neurophysiological measures, such as cortical excitability and biomarkers of neuroplasticity, could provide deeper insights into the mechanisms underpinning the observed functional outcomes.

## 7. Conclusions

The current study demonstrates that combining anodal tDCS with balance training selectively enhances endurance performance, but not balance, gait, or leg strength, in healthy older adults. While both the intervention and control groups benefited from exercise, the additional gains in 6MWT performance suggest that tDCS may augment exercise-induced improvements in domains sensitive to aerobic or functional capacity. These findings have meaningful implications for the use of neuromodulation in aging populations, where maintaining physical endurance is crucial for independence and quality of life. Importantly, this study underscores the need to refine and personalize tDCS protocols. Parameters, such as stimulation timing, intensity, electrode placement, and application mode, should be systematically optimized. Future research should explore the effects of online stimulation, particularly during task execution, and assess whether the combination of tDCS and exercise can yield sustained improvements with long-term follow-up. Studies targeting clinical populations with impaired plasticity, as well as incorporating neurophysiological and biomarker analyses, may provide deeper insight into the underlying mechanisms and broader applicability of combined tDCS-exercise interventions.

## Figures and Tables

**Table 1:** Demographic characteristics of participants.

		Group A	Group B
		(n = 15)	(n = 15)
Age (years)			
	Mean, SD	71.73, 5.80	74.87, 7.42
	Range	(65–85)	(65–90)
Body mass index (kg/m^2^)			
	Mean, SD	23.84, 3.28	23.03, 2.89
	Range	(18.73–28.89)	(18.73–28.89)
Gender, n (%)			
	Male	1 (6.67)	5 (33.33)
	Female	14 (93.33)	10 (66.67)
Waist and hip ratio			
	Mean, SD	0.90 ± 0.06	0.91 ± 0.08
	Range	0.86–0.93	0.86–0.96
Number of medications, n (%)			
	No	7 (46.67)	5 (33.33)
	1	4 (26.67)	4 (26.67)
	2	2 (13.33)	2 (13.33)
	≥3	2 (13.33)	4 (26.67)
Underlying disease, n (%)			
	No	7 (46.67)	5 (33.33)
	Diabetes Mellitus	2 (13.33)	3 (20)
	Hypertension	6 (40)	6 (40)
	Hyperlipidemia	2 (13.33)	3 (20)
	Other	7 (46.67)	3 (20)

Participants underwent anodal transcranial direct current stimulation (tDCS) thrice weekly, in conjunction with balance exercises over a six-week period. Group A received anodal tDCS, while Group B received sham tDCS.

**Table 2:** Comparative analysis of outcomes between Group A and Group B over the same timepoints.

Outcomes	Group A (n = 15)		Group B (n = 15)		Between Group Difference				
					Mean	SE	95% CI		*p*-Value
	Mean	SD	Mean	SD			Lower	Upper	
**Timed Up and Go Test**									
Baseline	10.89	9.29	11.12	1.89	−0.23	0.71			
Post-intervention	9.29	1.31	10.53	1.92	−1.23	0.62	N/A	N/A	N/A
Four weeks post-intervention	9.89	1.43	10.84	2.53	−0.96	0.78			
**One-Leg Stand Test**									
Baseline	16.89	11.54	20.88	21.87	−4.00	6.61			
Post-intervention	25.34	17.25	23.65	20.26	1.69	7.11	N/A	N/A	N/A
Four weeks post-intervention	24.31	18.06	22.05	17.10	2.26	6.65			
**Lower-Limb Strength Test**									
Baseline	50.40	22.00	54.13	21.76	−3.73	8.27			
Post-intervention	69.20	25.33	71.87	24.41	−2.67	9.40	N/A	N/A	N/A
Four weeks post-intervention	65.67	20.50	62.67	19.21	3.00	7.51			
**Six-Minute Walk Test**									
Baseline	294.67	47.19	293.27	46.09	1.40	17.63	−34.71	37.51	0.937
Post-intervention	361.73	50.20	357.67	58.38	4.07	21.19	−39.33	47.46	0.849
Four weeks post-intervention	354.20	50.20	314.00	54.11	40.20	19.73	−0.21	80.61	0.051

Group A received 2 mA of anodal transcranial direct current stimulation (tDCS) for 20 min, three times a week, in conjunction with balance exercises over a six-week period. Group B underwent sham tDCS along with the same balance exercises and duration. A *p* value < 0.05 indicates statistical significance between groups, as determined by the post-hoc analysis of the repeated ANOVA.

**Table 3:** The differential outcomes within the group observed at baseline, post-intervention, and follow-up.

Outcomes		Group A (n = 15)					Effect Size	Group B (n = 15)					Cohen’s d
		Mean	SD	95% Confidence Interval		*p*-Value		Mean	SD	95% Confidence Interval		*p*-Value	Effect Size
				Lower	Upper					Lower	Upper		
**Timed Up and Go Test**													
	Baseline-Post Intervention	1.59	0.34	0.67	2.51	0.001 **	1.03	0.59	0.15	0.19	0.99	0.004 **	0.32
	Baseline-Follow up	1.00	0.42	−0.13	2.13	0.092	0.62	0.27	0.28	−0.49	1.04	1.000	0.13
**One-Leg Stand Test**													
	Baseline-Post Intervention	−8.45	2.37	−14.88	−2.02	0.009 **	0.60	−2.77	0.98	−5.43	−0.11	0.041 *	0.14
	Baseline-Follow up	−7.42	2.83	−15.12	0.28	0.061	0.51	−1.17	3.20	−9.86	7.53	1.000	0.06
**Lower-Limb Strength Test**													
	Baseline-Post Intervention	−18.80	3.20	−27.49	−10.12	<0.001 ***	0.82	−17.73	3.41	−27.00	−8.47	<0.001 ***	0.79
	Baseline-Follow up	−15.27	2.94	−23.27	−7.27	<0.001 ***	0.74	−8.53	2.36	−13.59	−3.48	0.003 **	0.34
**Six-Minute Walk Test**													
	Baseline-Post Intervention	−67.07	11.73	−98.95	−35.19	<0.001 ***	1.37	−64.40	11.74	−96.30	−32.50	<0.001 ***	1.27
	Baseline-Follow up	−59.53	11.35	−90.37	−28.70	<0.001 ***	1.26	−20.73	12.40	−54.44	12.97	0.350	0.43

Group A received 2 mA of anodal transcranial direct current stimulation (tDCS) for 20 min, three times a week, in conjunction with balance exercises over a six-week period. Group B underwent sham tDCS along with the same balance exercises and duration. * represents statistical significance at *p* < 0.05, ** represents statistical significance at *p* < 0.01, and *** represents statistical significance at *p* < 0.001, as determined by the post-hoc analysis of the repeated ANOVA. Effect size was determined by Cohen’s d: 0.2 = small effect size, 0.5 = medium effect size, 0.8 = large effect size.

## Data Availability

Data are available on request due to privacy restrictions.
